# Development of a strategy for the expansion of online teaching at the University of Würzburg based on the experiences of lecturers and students in the pandemic years 2020/21

**DOI:** 10.3205/zma001667

**Published:** 2024-02-15

**Authors:** Lisa Marie Kühl, Nina Luisa Zerban, Elena Tiedemann, Sarah König

**Affiliations:** 1 University Hospital Würzburg, Institute of Medical Teaching and Medical Education Research, Würzburg, Germany; 2 University Hospital Würzburg, Department of General Practice, Würzburg, Germany

**Keywords:** distance education, medical education, teaching, digitalisation, COVID-19, Blended Learning, Constructive Alignment

## Abstract

**Background:**

Owing to the COVID-19 pandemic, the summer of 2020 saw face-to-face teaching replaced by online teaching. The question arose as to how digitalisation may be implemented meaningfully. The views of lecturers and students on past online programmes were gathered in order to identify potential and future prospects.

**Project description:**

An exploratory, guidelines-based interview study was conducted during the clinical phase of the medicine degree at the Faculty of Medicine in Würzburg. Five lecturers and five students were interviewed in the winter semester of 2020/21. This was followed by a content analysis evaluation according to Kuckartz, with the help of MAXQDA.

**Results:**

Online teaching offers more flexibility and security for the future. Hybrid formats (e.g., blended learning) are in demand. While theoretical knowledge can be taught online, face-to-face teaching remains essential in practical training. Digital elements must be developed didactically and anchored in the curriculum. Interaction and direct feedback between students and lecturers are key aspects of this.

**Discussion:**

Online teaching in medicine offers numerous potentials and didactic design options that can improve the degree programme in a competency-based manner. Combined teaching formats are particularly effective in this regard. Fittingly conceived, multimedia teaching formats enable students to approach their studies in a focused manner. The points raised during the interviews correspond with the fundamental principles of the ARCS model, which was developed to strengthen continuous motivation in students.

**Conclusion:**

Well-thought-out design and integration of online teaching can contribute to attractive, efficient, and future-oriented teaching/learning activities. Decisive factors are the collaboration of everyone involved and adequate provision of both time and financial resources.

## Zusammenfassung

**Hintergrund:**

Aufgrund der COVID-19-Pandemie wurde die Präsenzlehre im Sommer 2020 durch Online-Lehre ersetzt. Es stellte sich die Frage, wie die Digitalisierung sinnvoll implementiert werden kann. Die Sichtweise Dozierender und Studierender auf vergangene Online-Angebote wurde erhoben, um Potentiale und Zukunftsaussichten zu identifizieren.

**Projektbeschreibung:**

Eine explorative, leitfadengestützte Interview-Studie wurde im klinischen Abschnitt des Humanmedizinstudiums an der Medizinischen Fakultät Würzburgs durchgeführt. Je fünf Dozierende und Studierende wurden im Wintersemester 2020/21 befragt. Es folgte die inhaltsanalytische Auswertung nach Kuckartz unter Zuhilfenahme von MAXQDA.

**Ergebnisse:**

Online-Lehre bietet mehr Freiheitsgrade und Sicherheit für die Zukunft. Mischformate (z.B. Blended Learning) sind gefragt. Während theoretisches Wissen online vermittelt werden kann, ist die Vor-Ort-Präsenzlehre für die praktische Ausbildung unerlässlich. Digitale Elemente sind didaktisch auszuarbeiten und curricular zu verankern. Dabei sind die Interaktion und das direkte Feedback zwischen Studierenden und Dozierenden Schlüsselaspekte.

**Diskussion:**

Online-Lehre in der Medizin bietet zahlreiche Potenziale und didaktische Gestaltungsmöglichkeiten, die das Studium kompetenzbasiert verbessern können. Dabei sind kombinierte Lehrformate besonders wirksam. Passgenau konzipierte, multimediale Lehrformate ermöglichen den Studierenden eine fokussierte Herangehensweise. Die während der Befragungen genannten Punkte korrespondieren mit den Grundprinzipien des ARCS-Modells, das entwickelt wurde, um die durchgängige Motivation der Studierenden zu stärken.

**Schlussfolgerung:**

Eine gut durchdachte Gestaltung und Einbettung von Online-Lehre kann zu attraktiven, effizienten und zukunftsorientierten Lehr-/Lernaktivitäten beitragen. Entscheidend ist die Zusammenarbeit aller Beteiligten sowie eine angemessene Ausstattung mit zeitlichen und finanziellen Ressourcen.

## 1. Introduction

Despite the global advancement of digitalisation, there were hardly any online teaching formats in medical education in Germany that were anchored in the curriculum and established across faculties until the outbreak of the COVID-19 pandemic. Most of these were voluntary courses [1]. With the spread of COVID-19 and infection control measures, university teaching changed abruptly. After converting on-site face-to-face teaching to online formats in the summer semester of 2020 as an emergency solution, the question arose from the winter semester of 2021/22 on as to how these could be implemented in a didactically meaningful and needs-oriented manner. In this process, online teaching formats evolved into a sustainable and high-quality form of teaching for medical professionals of the future [2]. However, in doing so, it was desirable to develop an evidence-based strategy for online teaching that supports students and lecturers alike.

Even before the pandemic, Haag et al. observed that “[modern] teaching and learning technologies (...) are becoming increasingly important, especially in the context of blended learning concepts” [3]. Here, on-site face-to-face teaching and online teaching formats should be combined in a meaningful way so that teaching is organised as efficiently as possible [3]. As early as 2008, Cook et al. showed that integrating online teaching into traditional formats and traditional formats alone were at least equivalent in terms of learning success [4]. Liu et al. confirmed in 2016 that blended learning programmes have a positive effect on learning success. Compared to traditional formats, blended learning was at least on a par with traditional teaching formats. In some cases, learning success was even higher [5]. Nevertheless, Liu et al. emphasized that these conclusions should be treated cautiously, as they are subject to the heterogeneity in teaching [5]. In this context, Dziuban et al. emphasised that the development of blended learning is directly linked to the general technological advancements of the current era [6].

Prior to the pandemic even, the acceptance and understanding of the usefulness of blended learning concepts improved over the years. However, it was previously unclear as to what specific strategies should be followed in the implementation of blended learning [7]. In their post-pandemic study, Marques-Sule et al. revealed that blended learning can improve both knowledge and skills as well as perception and satisfaction of students. Thus, blended learning represents a step in the right direction towards innovative teaching formats. Nevertheless, it remains unclear under which conditions and in what form blended learning can be effectively integrated into teaching, as location- and subject-specific circumstances must be taken into account [7].

To develop an evidence-based strategy for online teaching, it was necessary to evaluate the views and experiences of lecturers and students on past online teaching. The aim was to identify advantages and disadvantages as well as optimisation approaches. Participants were surveyed with regard to their perspectives on everyday teaching/learning during COVID-19, aiming to derive potentials that demonstrate the development towards didactically reworked teaching [3], [8], [9]. The overarching target of the study was to contribute to the “improvement of teaching” [10].

Teaching can be considered to be of high quality when the people involved approach and emerge from it empowered and motivated. It was thus necessary to determine which factors particularly motivate students and encourage them to participate in teaching formats. In 2006, Astleitner presented the ARCS model according to Keller and Kopp, which introduces the promotion of motivation in the context of online teaching [11]. This comprises four key concepts intended to maximise student motivation (see table 1 (Tab. 1)). To develop an evidence-based strategy that integrates blended learning as a teaching format into medical education, the ARCS model may be used as a guide. 

For example, teaching formats should also be checked to see if they attract students' attention and convey relevant content. If this is the case, students are likely to be more motivated towards the teaching formats.

The following questions regarding the perspectives of students and lecturers were analysed in the context of this interview study and in view of the ARCS model:

- What potentials arise from the digitalisation of teaching during the COVID-19 pandemic?
- How can online teaching formats beneficially complement future teaching?

## 2. Project description

An exploratory guidelines-based interview study was conducted at the Julius-Maximilians-University in Würzburg. This involved collecting the subjective perceptions of lecturers and students during the clinical phase of the medicine degree at the Faculty of Medicine regarding online teaching in the summer semester of 2020 and winter semester of 2020/21. A qualitative study design was chosen to capture, interpret, and link subjective impressions individually.

Data were collected using semi-structured interviews (see attachment 1 ). Open questions set a certain direction without influencing the extent or personal evaluation of individual statements. The aim was to record subjective advantages and disadvantages, as well as individual approaches to studying and teaching during the COVID-19 pandemic. In addition, the aim was to enquire about wishes and suggestions regarding future teaching as well as identify obstacles and limitations.

The institutional review board of the University of Würzburg reviewed and approved an application to conduct the interview study (Processing No. 20201204 01). Potential interviewees were informed about the general conditions and study objectives in a cover letter and invited to participate. The letter also served as a consent and data protection declaration. It assured that no conclusions could be drawn about individuals or statements made in the interviews. Personal data were stored separately from the interview data.

The interviewees were five lecturers with clinical teaching experience ranging from four to 17 years, and five students from the first five clinical semesters. The lecturers were selected from among the awardees of the special teaching award for online teaching [12]. The students were recruited via the student representatives. Demographic information of the interviewees is listed in table 2 (Tab. 2). 

Data collection took place between November 2020 and January 2021. The interviews were conducted online via Zoom^TM^ to comply with hygiene regulations. As a precaution, an audio track was also recorded with a voice recorder in case of any technical problems. The audio tracks were then transcribed word for word using the software package f4 and anonymized. This ensured that no conclusions could be drawn about personal information, locations, or institutes. For example: “@My colleague Mr./Ms. Name@ as a @specialist@ in internal medicine works as a private lecturer in @city@ at the @university@ and had to prepare a lot of teaching material”.

The content-structuring qualitative content analysis was carried out on the anonymized transcripts according to Kuckartz [13] with the help of MAXQDA [14]. The aim was to create a category handbook with which the interview material was to be coded. Initially, the material was inductively divided into broad categories. These were based on the interview topics and the interview guidelines themselves. Subsequently, the main categories were stepwise differentiated into subcategories with up to six levels. Each category was defined and provided with an anchor example. The category handbook was then shortened by combining identical categories. Multiple responses by a person within a category were counted multiple times.

In this study, the core themes “general potentials of online teaching” and “online teaching of the future” were highlighted. For clarity purposes, statements made by students were marked with “S” and those made by lecturers with “D”. The subsequent number denotes the interview number.

## 3. Results

The categories listed in the tables are described in more detail in the results section and the following discussion if mentioned three or more times. Key aspects of the interviews are thus brought to the fore. 

### 3.1. General potentials of online teaching 

This main category describes all the elements perceived as advantageous by the interviewees. It concerns the potential benefits of online teaching. Table 3 (Tab. 3) presents the analysed statements separated according to the interview groups. 

#### 3.1.1. Students 

Students primarily described the many degrees of freedom as beneficial. The elimination of numerous on-site face-to-face teaching sessions saved travelling time (S1; S3-5), which could then be reinvested in studying or used otherwise. Asynchronous online teaching offered freedom in time management, allowing students to coordinate the processing of content independently (S1; S5). The permanent availability of material in the form of online lectures could be seen as a sustainable investment for future semesters (S1; S5). Equally important was the multimediality established through online teaching (S1; S4; S5). Various technical approaches and didactic implementations made online teaching particularly appealing. Students were motivated to participate in teaching actively (S4). Multimediality was in line with the zeitgeist of a modern digital society, hence the necessity of its promotion (S5). 

#### 3.1.2. Lecturers 

Lecturers also believed that online teaching allowed a great deal of freedom for students (D8-10) and was sustainable (D8; D9). They viewed multimedia and online teaching as a form of individualism. A wide range of offerings could cater to different learning preferences. A large number of students could thus find teaching formats that suited them (D7; D8; D10). Against this background, lecturers assumed that students would achieve greater learning success, as they would learn individually using different materials and at their own pace (D8). They highlighted the potential of the principle of the inverted classroom. This refers to (online) self-study as preparation for on-site face-to-face teaching, which then serves to consolidate content and practical training [1], [15]. Acquisition of theory through online teaching formats by means of self-study was the best possible preparation for face-to-face practical training on-site (D6; D8). The overarching principle of blended learning was categorised as particularly effective. This describes the sensible combination of online teaching formats and on-site face-to-face teaching [1], [3]. Inverted classrooms could be part of this to create significant valences (D6-8). This required curricular coordination and integration of teaching formats. Competence-based theory acquisition and practical skills would thus be promoted. On-site face-to-face teaching should thus be used primarily to acquire practical skills, which should take place in contact with lecturers and patients. Theoretical knowledge may be acquired digitally through asynchronous self-study or synchronous online teaching formats (D7-9).

### 3.2. Online teaching of the future 

This main category describes the online teaching to be implemented in the future based on the ideas of the interviewees. Attachment 2 summarises the statements of the interviewees. Owing to the similarity between the statements and the common design of the future of online teaching, no distinction was made between the perspectives of the interview groups. A joint presentation was thus made. 

#### 3.2.1. Desires for upcoming semesters – didactically and organisationally 

Regarding didactic wishes, interviewees raised the possibility of supplementing on-site face-to-face teaching with modified online teaching formats. Practical components (e.g., patient presentations) should take place on site and face to face (S2-4; D8). Theoretical modules could be realized for example through online lectures and discussion rounds (S4-5; D8). Generally, especially with online teaching formats, support from lecturers was of significance (S2). Interviewees would like to have recordings of synchronous online teaching formats (S5) or streaming of lectures taking place on site and face to face (S5; D8; D10). Overall, it emerged that both interview groups favoured a combination of online and on-site teaching (e.g., inverted classrooms) and would like to see this in the future (S1; S3-5; D6-10). Regarding organizational wishes, interviewees viewed the technical implementation of teaching in terms of material creation (S5; D7-9) and provision as in need of improvement (S3). Both feedback from students and lecturers' ability to accept criticism were important for improving the overall situation (S2; D8-10). There was a consensus that communication between all parties is central to high-quality, sustainable teaching (S2; D6-10). 

#### 3.2.2. Combining good teaching and online teaching 

Interviewees agreed that good teaching and online teaching could go hand in hand (S1; S2; S4; S5; D7; D8). Specifically, the combination of online and on-site teaching reflected a form of good teaching (S1; S3; D6; D7; D9; D10). Generally, good online teaching was feasible (S5; D8). Overall, the focus should be on subject-specific teaching design. Each bit of teaching content required an individual format to be conveyed with high quality (S2; S4; D7; D10). Combined teaching formats and small-group classes would make it possible to meet the individual learning needs of students (D7; D8). 

#### 3.2.3. Limits of online teaching 

Interviewees criticized the limited preparation towards professional practice. The omission of on-site face-to-face practical and bedside teaching could not be compensated for online. At least some on-site face-to-face teaching had to be reintroduced (S2; S4; D7; D9). 

#### 3.2.4. Political framework 

Investment in time resources proved to be necessary (D8). The healthcare system needed to be improved to better integrate teaching into the schedule of everyday clinical practice, ensuring adequate training for young medical students (D8). For this, educational policy would have to see to expanding resources (D8).

## 4. Discussion

### 4.1. The general potentials of online teaching

This study demonstrated that online teaching in medicine offers many potentials and didactic design options and make things easier in the future. Interviewees described online teaching unanimously as individual, free, versatile, accessible, and sustainable. As previously reported results indicate, it is a beneficial teaching format in medical education, helping to improve the degree course in a competency-based manner [8], [16], [17]. It was pointed out that online teaching is only successful when well-planned concepts are integrated into the curriculum. Online teaching also creates valences [8], [10], [18]. Blended learning concepts, for example, offer a high degree of freedom. Lecturers gain autonomy in design, and students have the opportunity to accept individualised courses. Accordingly, these combined teaching formats should be retained [8], [19]. Theoretical acquisition and competency training become possible through freed valences, especially if the principle of the inverted classroom is taken into consideration [15], [20], [21], [22]. Our study and the literature suggest this may lead to greater learning success and justify continuation [4], [5], [7], [22]. In terms of the ARCS model, it has been shown that the design of the teaching in particular fosters student motivation. Multimedia, purposefully developed teaching formats facilitate a focused approach to studying [1], [15], [19]. Ideally, online teaching should give students satisfaction and the feeling of having learned something important and useful. They should have the impression that their efforts are rewarded with success. This boosts confidence and increases learning motivation. Online teaching should not be seen as standalone and independent, but requires a clever combination with on-site teaching in the sense of blended learning. It is clear that lecturers must create online teaching formats very precisely and update them regularly. This requires a teaching effort that must be adequately measured [19]. 

### 4.2. Online teaching of the future

This study reveals that, for the location, teaching methods in medical education need to be further adapted for the future. An appropriate evidence-based strategy is important for the implementation of online teaching formats. Only through coherent teaching organization and coordination can their potentials be exploited fully. It is therefore necessary to reduce organisational deficits. Teaching must also consider both the individual needs of students as well as the subject-specific requirements. As other studies indicate, the didactic design of teaching formats should particularly focus on relevant topics [19]. Lecturers need support in implementing the curriculum (e.g., in determining the scope of timing and content) [10], [22]. In line with other studies, interdisciplinary exchange and direct feedback from students are crucial for the design of the overall teaching concept [10], [15], [19], [21]. Although online education has weaknesses teaching practical skills, combined teaching formats such as the inverted classroom prove to be a good addition to traditional medical training even beyond the COVID-19 pandemic [23]. High-quality teaching results when all stakeholders collaborate and take individual needs into consideration [8], [18], [19]. Shortcomings in online teaching can be minimized if practical courses are held alongside digital offerings [8], [21], [23], [24]. To meet these didactic requirements, the support of education policy is crucial [10], [16], [23]. This requires sufficient time and financial resources. Based on the ARCS model, it can be summarized that high-quality teaching that is tailored to students and the subject area, and emphasises relevant content, increases the motivation to learn. This includes combined teaching formats and catering to individual needs [1], [3], [18], [21], [22]. It is important to provide students with adequate support during teaching and give them sufficient attention. Personal dialogue to clarify individual problems fosters student motivation. Despite their responsibility, they appreciate the interaction with lecturers [19].

## 5. Conclusions

Based on the latest findings in teaching and learning research, Schultz-Pernice et al. formulated specific guidelines for lecturers that pertain to evidence-based digital teaching and learning [19]. These recommendations are consistent with the results of our study and highlight the key elements of online teaching formats. Thus, we can summarize the following suggestions from our findings: Open communication between all involved parties is essential. This includes students, lecturers, and teaching coordination personnel, as well as exchanges at (inter-)faculty level and with bodies of educational policy. Moreover, face-to-face contact is necessary, especially since practical skills cannot be conveyed exclusively through online teaching. This study reveals that, despite all technological advancements, on-site face-to-face teaching, for example to teach skills, remains necessary and very much in high demand. It is important to structure learning materials sensibly and focus on relevant content. The overarching learning objective should be clear. Additionally, it helps if the teaching is not only convincing in regard to content but also visually and in terms of multimedia. Knowledge assessments are an important component of online teaching to identify and address deficits and knowledge gaps in a timely manner. Regular feedback as a means of evaluation is a helpful tool. Lecturers should involve students actively in online teaching to facilitate collaborative learning and support self-reflection. It is important to encourage students to retrieve and apply their knowledge independently. Generally, online teaching should be designed in collaboration within the faculty and, if possible, between different universities. Shared experiences and adopted methods or concepts can significantly simplify one’s own teaching design [19]. Finally, it is advisable to consider these points to expand and optimize online teaching in the future, with an openness to new didactic approaches being crucial [19].

This study demonstrates that online teaching should be methodically well thought out and firmly anchored in the curriculum. General guidelines and quality criteria are necessary, but they should still allow lecturers some individual room for manoeuvre in their application. The aim is to develop teaching that focuses on competencies and practical skills, incorporates the blended learning concept, and fits coherently with on-site face-to-face teaching. In order to develop appropriate teaching formats, lecturers must be trained accordingly and given sufficient time to develop and update them. This is particularly valid if such services are included in their teaching workload. Students, in turn, need guidance and support to make the best possible use of the digital teaching programmes on offer.

The study has limitations. The study group is small and location-specific, which limits the representativeness of the study beyond the individuals and subjects surveyed in the degree course. Given the focus on the clinical phase of the degree course, preclinical semesters were not taken into account. Additionally, the interview study was conducted during the COVID-19 pandemic, so some interviewees may have adopted newer approaches by now. In conclusion, the strength of the study lay in the development of an evidence-based strategy for the Faculty of Medicine in Würzburg that did not exist in this form previously.

## Authors’ ORCIDs

- Lisa Marie Kühl: 0009-0005-4647-4521
- Nina Luisa Zerban: 0009-0003-7122-2946
- Elena Tiedemann: 0009-0003-2393-6020
- Sarah König: 0000-0003-4866-9881

## Competing interests

The authors declare that they have no competing interests. 

## Supplementary Material

Interview guide

Online teaching of the future

## Figures and Tables

**Table 1:**
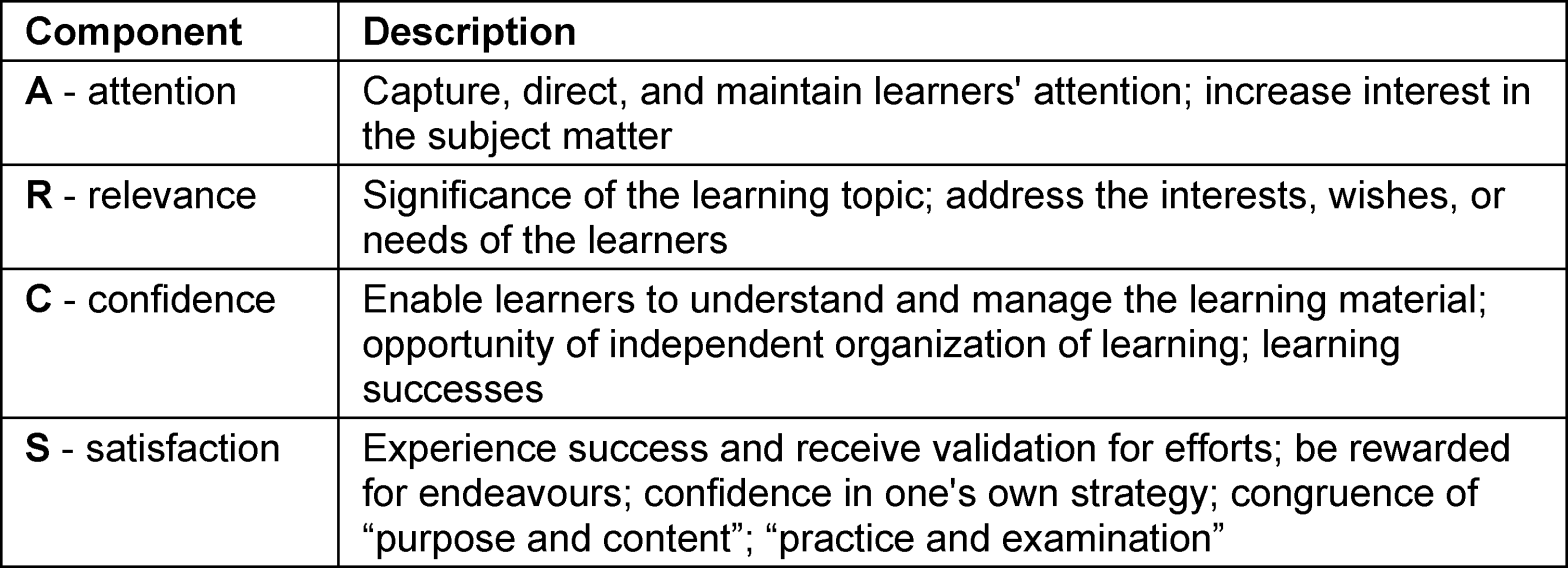
ARCS Model by Keller/Kopp, presented by Astleitner [11]

**Table 2:**
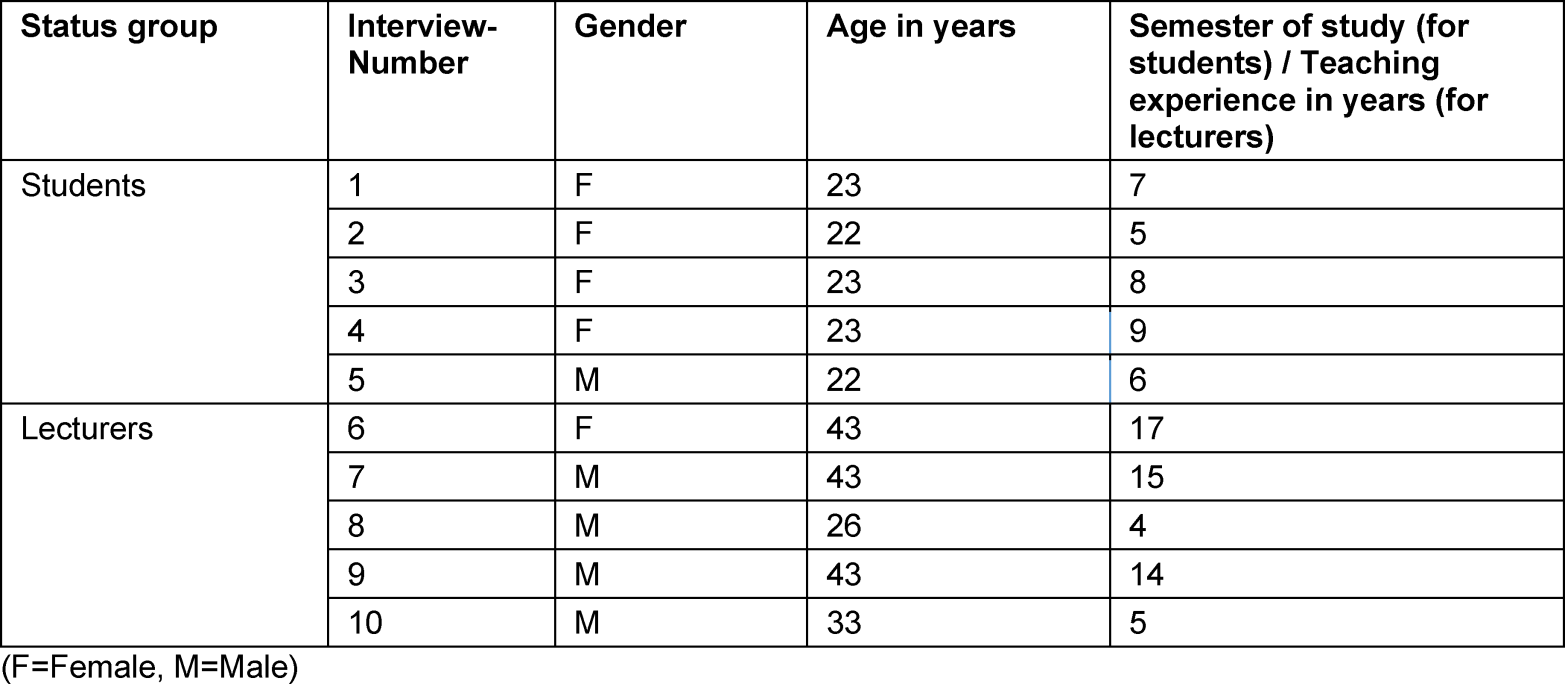
Interviewee demographics

**Table 3:**
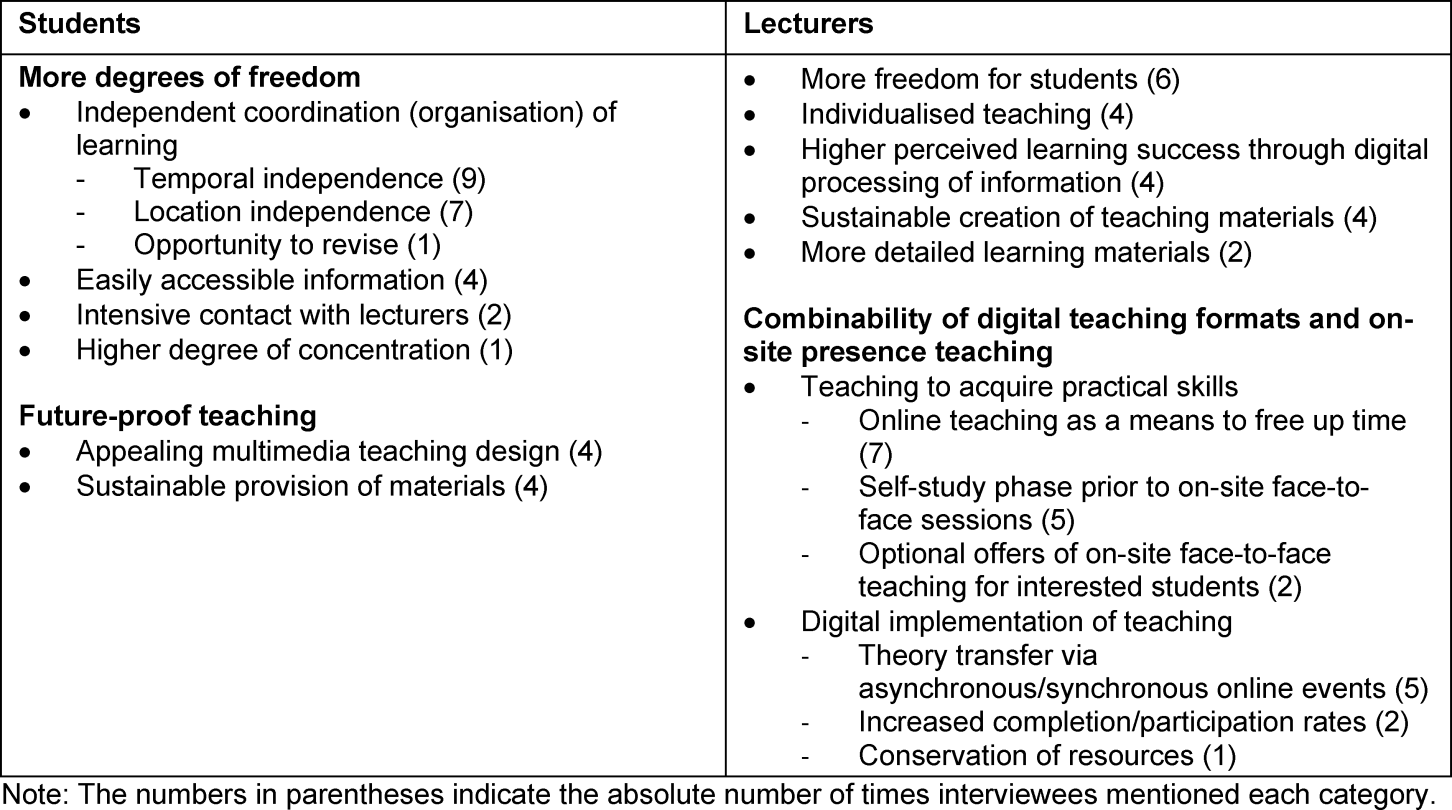
General potentials of online teaching
