# Supplementary material for: Development of a strategy for the expansion of online teaching at the University of Würzburg based on the experiences of lecturers and students in the pandemic years 2020/21
Source: GMS J Med Educ. 2024 Feb 15;41(1):Doc12. doi: 10.3205/zma001667 (PMC10946211; doi:10.3205/zma001667)
Supplement: Interview guide [file JME-41-12-s-001.pdf]

## Attachment 1: Interview Guide

**Supplementary questionnaire** for participation in guidelines-based interviews as part of the doctoral thesis by Lisa Marie Kühl

Age:

Gender:

Field of study/semester:

Teaching experience in years (of lecturers):

**Opening question:** How do you currently teach? -> *Has anything in your daily teaching routine changed?*

How do you currently learn? -> *Daily schedule? / Events?*

**Possible further prompts**

- How did you organise your teaching? / How did you perceive your teaching?
- What was different? Compared to “normal” semesters.

<table>
<tr><th>Lecturers</th><th>Question</th><th>Students</th></tr>
<tr><td colspan="3" align="center">Individual teaching/Learning design</td></tr>
<tr><td>→ How is teaching for you?</td><td>➔ How did you feel about teaching?<br>(preparation, follow-up, execution)<br>→ Inquire about challenges, if mentioned</td><td>➔ How was the learning experience for you?</td></tr>
<tr><td colspan="3" align="center">Effort in teaching organisation/Self-study</td></tr>
<tr><td></td><td>How much time did you invest?<br>- What in?<br>- Compared to previous semesters?</td><td></td></tr>
<tr><td colspan="3" align="center">Support for teaching</td></tr>
<tr><td></td><td>What support did you receive?<br>- Were there things you would have wished for explicitly? If yes, what and why?<br>- Follow-up question: personal/private<br>(material / infrastructure)</td><td></td></tr>
<tr><td colspan="3" align="center">Challenges</td></tr>
<tr><td></td><td>What were the challenges during this period?<br>- Which aspects were particularly neglected/would you have liked to do differently? Please give reasons for your answer<br>- What is online teaching particularly suitable for?</td><td></td></tr>
</table>

Attachment 1 to Kühl LM, Zerban NL, Tiedemann E, König S. *Development of a strategy for the expansion of online teaching at the university of Würzburg based on the experiences of lecturers and students in the pandemic years 2020/21.* GMS J Med Educ. 2024;41(1):Doc12. DOI: 10.3205/zma001667

| | | |
|---|---|---|
| Advantages and disadvantages | | |
| | ➔ Please name an advantage and disadvantage of online teaching relevant for you and justify your choice. | |
| Experiences/Assessments/Outlook | | |
| | Does good teaching conflict with online teaching?<br>- What exactly?<br>- Why? | |
| What experiences from teaching during the COVID-19 pandemic will you carry over to your future teaching? Why?<br>What will remain for you as a teacher in the future?<br>What would be an ideal mix between online teaching and on-site teaching?<br>- What *cannot* be digitised at all? | What do you wish for the future?<br>- Why?<br>(teaching/learning activities) | Based on the experiences of the semesters during the COVID-19 pandemic, what do you wish for your further studies? Why?<br>What will remain for you as a student in the future?<br>What would be an ideal mix between online teaching and on-site teaching?<br>- What *cannot* be digitised at all? |
| Further prompts | | |
| | | Do you think you have any advantages or disadvantages compared to other students?<br>How prepared do you feel for the exams? |
| Please emphasise **the** potential again.<br>*-> possibly a specific advantage and disadvantage* | | |
| | OPTIONAL: personal motivation | |
| OPTIONAL: Why did you get involved? (involvement/participation) | | |
