# Supplementary material for: Development of a strategy for the expansion of online teaching at the University of Würzburg based on the experiences of lecturers and students in the pandemic years 2020/21
Source: GMS J Med Educ. 2024 Feb 15;41(1):Doc12. doi: 10.3205/zma001667 (PMC10946211; doi:10.3205/zma001667)
Supplement: Online teaching of the future [file JME-41-12-s-002.pdf]

Attachment 2: Online teaching of the future

**Wishes for the upcoming semesters**		**Combinability of good teaching and online teaching**	**Limits of online teaching**	**Political framework**
**Didactic**	**Organisational**			
**On-site face-to-face**<br>• Link between theory and practice (19)<br>• Discussion of patient presentations / examples (10)<br>• Catch up on missed lessons (2)<br>• Return to in-person lectures (2)<br>**Digital format**<br>• Retain digital lectures (12)<br>• Opportunity to discuss questions synchronously (7)<br>• Support from teachers (4)<br>• Desire for videos of synchronous events (4)<br>• Increased case-based study (1)<br>• Greater familiarity with new digital concepts (1)<br>• Provision of materials collectively at the beginning of the semester (1)<br>• Expansion of the existing digital repertoire (2)	• Assistance in teaching design from the university<br>- Medical-didactic exchange (4)<br>- Increased capacities of university platforms (2)<br>• Explicit verbal feedback (5)<br>• Clearly structured course rooms on learning platforms (4)<br>• Timely provision of materials (3)<br>• Improved technical implementation of teaching on offer (2)<br>• More planning security regarding semester course (2)<br>• Training for lecturers in didactics of online teaching (1)<br>• Coordination among subjects regarding study load (1)<br>• Need for alignment of existing teaching content (1)	• Concepts of mixing digital and on-site face-to-face teaching (6)<br>• Feasibility of good online teaching (6)<br>• Individual teaching concepts for each subject (4)<br>• Student-friendly teaching (3)	• Lack of preparation for professional qualification<br>- Insufficient acquisition of practical skills (6)<br>- Lack of sustainability of acquired knowledge (1)<br>• Lack of guided introduction to new areas of teaching (1)<br>• Not addressing various learning preferences (1)	• Expansion of time resources for teachers (3)<br>• Expansion of financial resources (2)<br>• Support from education policy (2)

Attachment 2 to Kühl LM, Zerban NL, Tiedemann E, König S. *Development of a strategy for the expansion of online teaching at the university of Würzburg based on the experiences of lecturers and students in the pandemic years 2020/21.* GMS J Med Educ. 2024;41(1):Doc12. DOI: 10.3205/zma001667

**Mix of on-site teaching and digital concepts**<br>• Inverted classroom to prepare for on-site face-to-face courses (9)<br>• Dualism in teaching implementation (8)<br>• Optional in-person attendance in lectures with online streaming (4)<br>**Individuality**<br>• Personal responsibility regarding mental health (2)<br>• Multimodal teaching (1)<br>• Subject-specific teaching design (1)				

Note: The numbers in parentheses indicate the absolute number of times interviewees mention each category.
